# A *PAX1* enhancer locus is associated with susceptibility to idiopathic scoliosis in females

**DOI:** 10.1038/ncomms7452

**Published:** 2015-03-18

**Authors:** Swarkar Sharma, Douglas Londono, Walter L. Eckalbar, Xiaochong Gao, Dongping Zhang, Kristen Mauldin, Ikuyo Kou, Atsushi Takahashi, Morio Matsumoto, Nobuhiro Kamiya, Karl K. Murphy, Reuel Cornelia, TSRHC Scoliosis Clinical Group, L. Karol, K. Rathjen, D. Sucato, J. Birch, C. Johnston, B. S. Richards, T. Milbrandt, V. Talwakar, H. Iwinski, R. Muchow, J. C. Tassone, X. -C. Liu, R. Shindell, W. Schrader, C. Eberson, A. Lapinsky, R. Loder, J. Davey, Japan Scoliosis Clinical Research Group, Naobumi Hosogane, Yoji Ogura, Yohei Takahashi, Atushi Miyake, Kota Watanabe, Kazuhiro Chiba, Yoshiaki Toyama, Katsuki Kono, Noriaki Kawakami, Taichi Tsuji, Koki Uno, Teppei Suzuki, Manabu Ito, Hideki Sudo, Shohei Minami, Toshiaki Kotani, Haruhisa Yanagida, Hiroshi Taneichi, Ikuho Yonezawa, Kazuo Kaneko, John A. Herring, Dennis Burns, Nadav Ahituv, Shiro Ikegawa, Derek Gordon, Carol A. Wise

**Affiliations:** 1 Sarah M. and Charles E. Seay Center for Musculoskeletal Research, Research Department, Texas Scottish Rite Hospital for Children, Dallas, Texas 75219, USA; 2 Department of Genetics and Human Genetics Institute, Rutgers University, Piscataway, New Jersey 08854, USA; 3 Department of Bioengineering and Therapeutic Sciences, Institute for Human Genetics, University of California San Francisco, San Francisco, California 94143, USA; 4 Laboratory of Bone and Joint Diseases, Center for Integrative Medical Sciences, RIKEN, Tokyo 108-8639, Japan; 5 Laboratory for Statistical Analysis, Center for Integrative Medical Sciences, RIKEN, Yokohama 230-0045, Japan; 6 Department of Orthopaedic Surgery, School of Medicine, Keio University, Tokyo 108-8345, Japan; 7 Department of Orthopaedics, Texas Scottish Rite Hospital for Children, Dallas, Texas 75219, USA; 8 Department of Orthopaedic Surgery, University of Texas Southwestern Medical Center at Dallas, Dallas, Texas 75390, USA; 9 Department of Pathology, University of Texas Southwestern Medical Center at Dallas, Dallas, Texas 75390, USA; 10 McDermott Center for Human Growth and Development, University of Texas Southwestern Medical Center at Dallas, Dallas, Texas 75390, USA; 11 Department of Pediatrics, University of Texas Southwestern Medical Center at Dallas, Dallas, Texas 75390, USA; 12 Department of Orthopedic Surgery, Texas Scottish Rite Hospital for Children, Dallas, Texas, USA; 13 Department of Orthopaedic Surgery, Shriners Hospitals for Children, Lexington, Kentucky, USA; 14 Department of Orthopaedic Surgery, Children's Hospital of Wisconsin, Milwaukee, Wisconsin, USA; 15 OrthoArizona, Phoenix, Arizona, USA.; 16 Departments of Orthopedics, Sports Medicine, and Surgical Services, Akron Children's Hospital, Akron, Ohio, USA; 17 Pediatric Orthopaedics and Scoliosis, Hasbro Children's Hospital, Providence, Rhode Island, USA; 18 University of Massachusetts Memorial Medical Center, Worcester, Massachusetts, USA; 19 Indiana University-Purdue University Indianapolis, Indianapolis, Indiana, USA; 20 University of Oklahoma Health Sciences Center, Oklahoma City, Oklahoma, USA; 21 Department of Orthopaedic Surgery, Keio University, Tokyo, Japan; 22 Department of Orthopaedic Surgery, Eiju General Hospital, Tokyo, Japan; 23 Department of Orthopaedic Surgery, Meijo Hospital, Nagoya, Japan; 24 Department of Orthopaedic Surgery, National Hospital Organization, Kobe Medical Center, Kobe, Japan; 25 Department of Orthopaedic Surgery, Hokkaido University, Sapporo, Japan; 26 Department of Orthopaedic Surgery, Seirei Sakura Citizen Hospital, Sakura, Japan; 27 Department of Orthopaedic Surgery, Fukuoka Children's Hospital, Fukuoka, Japan; 28 Department of Orthopaedic Surgery, Dokkyo Medical University, Mibu, Japan; 29 Department of Orthopedic Surgery, Juntendo University School of Medicine, Tokyo, Japan

## Abstract

Idiopathic scoliosis (IS) is a common paediatric musculoskeletal disease that displays a strong female bias. By performing a genome-wide association study (GWAS) of 3,102 individuals, we identify significant associations with 20p11.22 SNPs for females (*P*=6.89 × 10^−9^) but not males (*P*=0.71). This association with IS is also found in independent female cohorts from the United States of America and Japan (overall *P*=2.15 × 10^−10^, OR=1.30 (rs6137473)). Unexpectedly, the 20p11.22 IS risk alleles were previously associated with protection from early-onset alopecia, another sexually dimorphic condition. The 174-kb associated locus is distal to *PAX1*, which encodes paired box 1, a transcription factor involved in spine development. We identify a sequence in the associated locus with enhancer activity in zebrafish somitic muscle and spinal cord, an activity that is abolished by IS-associated SNPs. We thus identify a sexually dimorphic IS susceptibility locus, and propose the first functionally defined candidate mutations in an enhancer that may regulate expression in specific spinal cells.

## Abstract (web summary)

Girls are tenfold more likely than boys to require surgical treatment for idiopathic scoliosis, a common paediatric skeletal disorder. Here, Sharma *et al*. identify the first sexually dimorphic idiopathic scoliosis risk locus, and demonstrate that it may play a role in the regulation of spinal cells.

## Graphical abstract

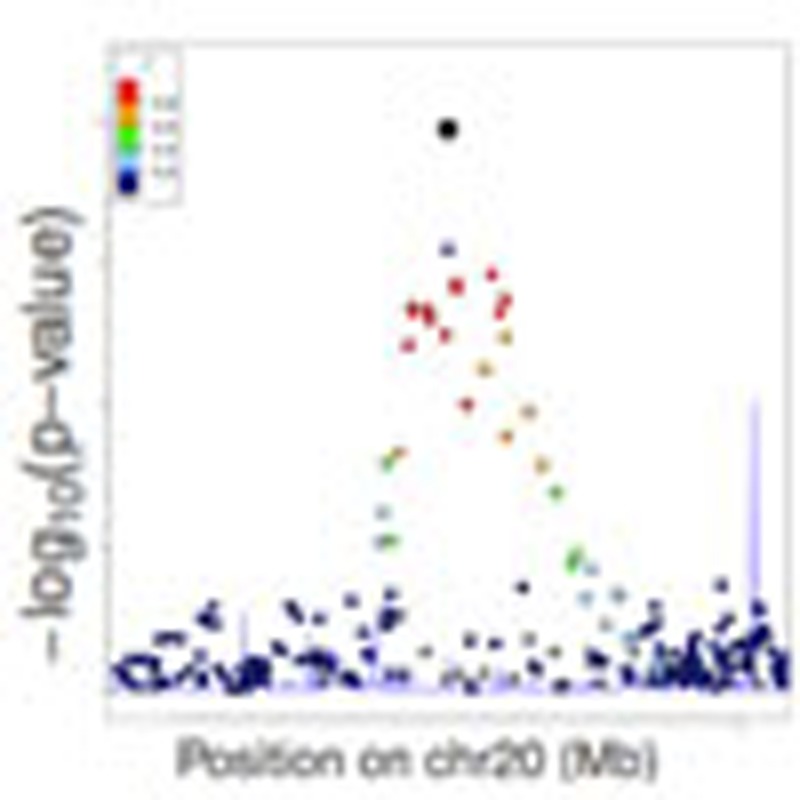

Scoliosis is defined as a curvature of the thoracolumbar spine greater than 10° in the coronal plane. Scoliosis is frequently secondary to other underlying diseases or seen as part of the phenotypic spectrum of heritable syndromes, in particular, disorders of neuromuscular or connective tissue development. In other patients, so-called ‘congenital’ scoliosis arises from frank malformations of the vertebrae that are most often isolated segmentation defects. However, for more than 80% of patients, the origins of scoliosis are unknown, occurring in individuals who are otherwise healthy and bear no obvious structural deficiencies in the vertebral column and associated soft tissues^1^. This ‘idiopathic’ scoliosis (IS) is the most common paediatric musculoskeletal disorder, affecting ~3% of children worldwide^2^. The onset of IS typically coincides with the adolescent growth spurt. Affected individuals are at risk for increasing deformity until growth ceases, although patients with large curves (>50°) may continue to worsen more slowly throughout adulthood^1^,^3^. Severe IS warrants surgical correction to prevent later disfigurement and other co-morbidities including back pain and pulmonary dysfunction^1^,^2^. Progression of the deformity can be rapid, prompting many states in the United States to require school screenings for early detection^4^.

Multiple biologic phenotypes are suspected in IS but have proven difficult to define, particularly as the involved structures, that is, the spine and associated soft tissues, appear superficially normal^5^. Functional and clinical assessments have associated IS with both neuropathologic/neuromuscular as well as connective tissue abnormalities^5^,^6^. These studies hint at multiple subphenotypes in human IS but have not defined causality. Aetiologic understanding of IS has also suffered from a lack of tractable, genetically defined animal models that clearly re-capitulate the phenotype. However, recent descriptions of IS-like phenotypes in both naturally occurring and genetically engineered teleosts hold out promise in this regard^7^. In particular, forward genetic and genome editing approaches in zebrafish (*Danio rerio*) have recently produced strains with an IS-like phenotype, suggesting that this system may be a powerful tool for modeling IS and defining its disease mechanisms^8^,^9^.

IS is a sexually dimorphic disease^10^. Girls and boys exhibit a striking difference in the prevalence of progressive IS, with girls having approximately tenfold greater risk of progressive curves that require operative treatment^11^. This dichotomy in female/male disease expression, and its correlation with the adolescent growth spurt have prompted investigations of hormonal influences in the development and progression of female IS^6^. Sexual dimorphism in IS has also been attributed to differences in genetic loading between males and females, with the least affected sex (males) requiring a stronger genetic load to acquire disease, a so-called Carter effect. One study of a cohort of multiplex families has provided epidemiological evidence for this genetic model^12^.

Genetic influences in IS were first suggested almost a century ago^5^. Segregation studies support a complex inheritance model in which multiple genetic factors contribute greater than 80% of the overall disease risk in IS^13^,^14^. Although most cases are sporadic, about 25% of IS patients report family history of IS, and more rare families with apparent Mendelian, or single-gene inheritance are described^15^. Early gene discovery efforts centred on applying traditional linkage mapping methods to search for causal genes in such extended pedigrees. These studies defined five IS candidate loci as noted in the Online Mendelian Inheritance in Man (OMIM): OS1 (OMIM 181800, chr19p13.3), OS2 (OMIM 607354, chr 17p11), OS3 (OMIM 608765, chr 8q12.1-12.2), OS4 (OMIM 612238, chr 9q31-q34) and OS5 (OMIM 612239, chr 17q25-qter). All but OS3 were defined in single extended pedigrees^16^,^17^,^18^,^19^. Common single-nucleotide polymorphisms (SNPs) for one candidate gene within OS3, *CHD7*, were significantly associated with IS in a cohort of 52 families^19^. Otherwise, the causal genes and mutations encoded within familial IS disease loci have not been forthcoming, most likely due to issues of genetic heterogeneity in IS that can confound traditional gene discovery approaches^20^.

As with other complex genetic disorders, the overall genetic architecture of IS is expected to reflect genetic factors with varying frequencies and effect sizes that will be discoverable by sequence- and haplotype-based methods^21^. Population-based genome-wide association studies (GWAS), a method only recently applied to IS, have proven powerful and efficient for mapping common susceptibility loci for hundreds of complex human traits^22^. Three published GWAS of IS have begun to define IS susceptibility loci^23^,^24^,^25^. A locus on chromosome 10q24.1 is the most studied and was initially identified by GWAS of 1,033 East Asian (Japanese) cases and 1,473 matched controls^24^. A recent combined analysis from multiple ethnic groups (that is, mostly East Asian and non-Hispanic white, NHW) provided further evidence for the locus, which is in the proximity of the *LBX1* gene (combined *P*=1.22 × 10^−43^ for rs11190870)^26^. *LBX1* encodes the ladybird homeobox 1 protein that is important for early muscle patterning as well as specification of dorsal horn neurons in developing spinal cord^27^,^28^,^29^. Causal mutations underlying this association and their potential effects on *LBX1* and/or other genes are not yet defined. A separate expanded analysis of the original East Asian GWAS yielded significant association with SNPs at a second locus, within the *GPR126* gene in chromosome 6, a result that was replicated in both East Asian and NHW cohorts^25^. *GPR126* encodes G-protein-coupled receptor 126 that is critical in early neurologic development and Schwann cell myelination^30^. It is interesting that SNPs in *GPR126* also have been associated with sitting height in humans^31^. In total, reported associations are estimated to explain less than 5% of the overall genetic contribution to disease risk. How these loci function in IS pathogenesis is as yet undefined^25^,^26^.

To discover new genetic risk factors for IS, we performed a two-stage GWAS in 3,102 individuals. Our results define a new susceptibility locus encoding associated SNPs that, surprisingly, are also associated with androgenic alopecia (AGA), or male pattern baldness. We find that the locus is specifically associated with female IS, suggesting that it contributes to the sexually dimorphic expression of the disease. By functional fine-mapping assays in zebrafish, we further define a sequence in the associated locus with enhancer activity that is abolished by IS-associated SNPs. Altogether, our results identify the first functionally characterized candidate mutations for IS susceptibility and expand our understanding of the role of non-coding regulatory elements in the disease. Our findings also suggest hypotheses to explain disease pathogenesis and provide the first insights into its puzzling sexual dimorphism.

## Results

### Association with common variants near the *PAX1* gene

**Figure 1:**
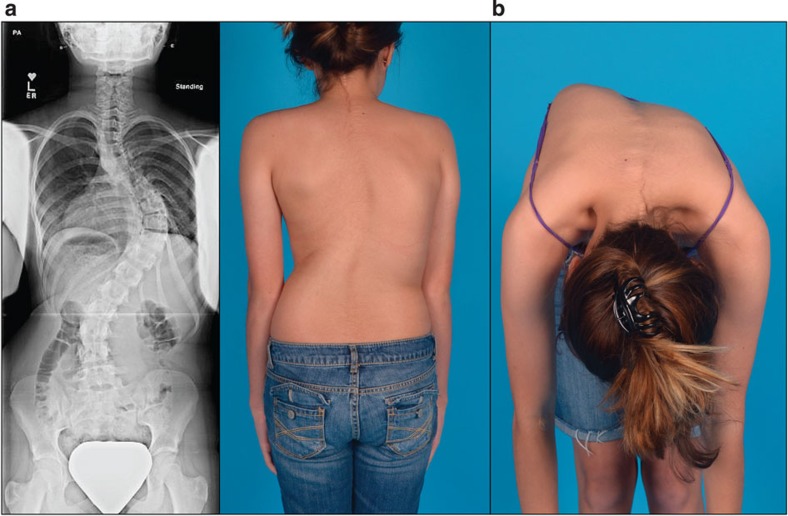
Idiopathic scoliosis in an adolescent female. (**a**) Lateral radiograph reveals prototypical right thoracic spinal curvature. Shoulder imbalance is evident in clinical photograph. (**b**) Prominent rib hump is evident on forward bending.

**Figure 2:**
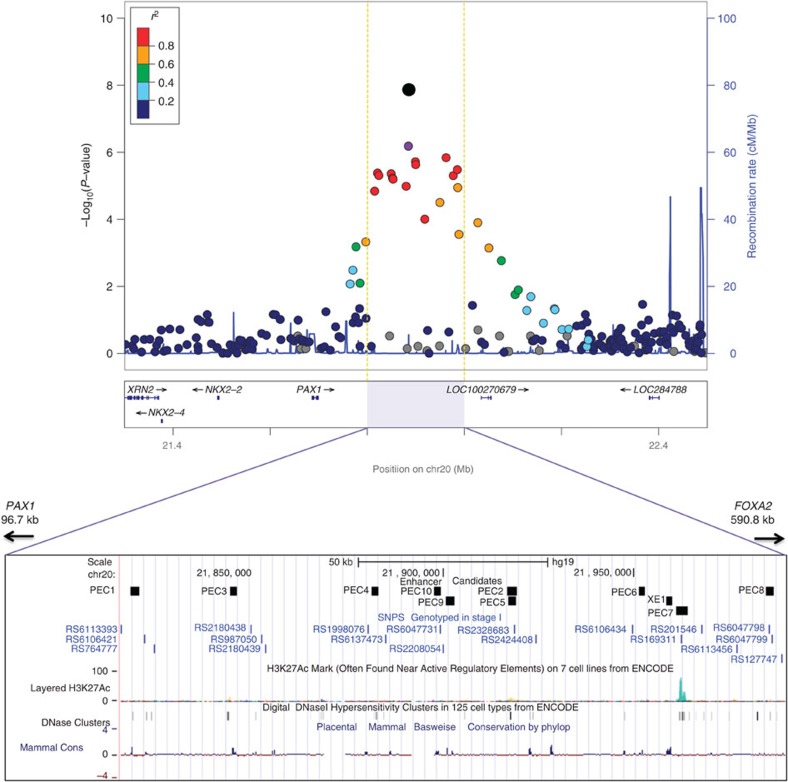
Chromosome 20p11.22 regional association plot. −Log 10 (*P*-value) of association with IS is plotted versus chromosomal position for each genotyped SNP for the second-stage GWAS (GWAS II). Each SNP is colour coded to reflect relative correlation (*r*^2^ value) with major SNP rs6137473 (purple dot). A physical map of the associated region is shown below the *x*-axis. The summary statistic *P*-value for the combined TSRHC GWAS (stages I and II) was calculated for the interval encompassed by yellow dotted lines as described in the text. This *P*-value is depicted by the black circle. Detailed view (shown below) of sequence conservation in the region highlighted in purple. Custom UCSC genome browser view with hg19 chromosome 20 positions of PEC and Xe1 sequence blocks and genotyped SNPs is shown. In the layered H3K27ac ENCODE track, the green represents HSMM (human skeletal muscle myoblast) cells.

Research subjects included in the two-stage GWAS were ascertained in Paediatric Orthopedic Clinics at the Texas Scottish Rite Hospital for Children (TSRHC). Affected individuals met standard criteria for a diagnosis of IS and had spinal deformity measuring at least 15° by the Cobb angle method (Fig. 1a,b). The GWAS I-715 included 715 trios from 702 trio families (parents and affected offspring, *N*=1,876 total) a portion of which was previously described^23^. The GWAS II case–control study included 482 independent IS-affected cases and 744 controls of self-reported NHW ethnicity that were independent of GWASI-715. Further ascertainment, genotyping, quality control and statistical methods are described in the Methods section. In the first stage, we genotyped GWAS II cases and controls using the Illumina HumanOmniExpress-12 v1.0 beadchip containing 730,498 markers. We used PLINK v1.07 (ref. 32 to perform quality assurance (QA) and test the data for association using the Cochran Armitage trend test (CATT). This analysis replicated the previously reported associations near *LBX1* and *GPR126* (refs 24, 25, 26). However, we found strongest results with SNPs rs6137473 (CATT, *P*=5.58 × 10^−7^, odds ratio (OR)=1.53, 95% confidence interval (CI)=1.29–1.80 for risk allele G) and rs169311 (CATT, *P*=1.25 × 10^−6^, OR=1.51, 95% CI=1.28–1.78 for risk allele A) in a region of chromosome 20p11.22 between the *PAX1* and *FOXA2* genes (Fig. 2). SNPs in the 20p11.22 inter-genic region remained the most significantly associated with IS after imputing additional genotypes for all of chromosome 20 (Supplementary Fig. 1). For the second stage, we expanded a prior GWAS^23^ from 419 to 715 parent-offspring trios (‘GWAS I-715’, 1,876 individuals). The ethnic composition of this cohort is given in Supplementary Fig. 2. All trios were analysed together using *TDT-HET*, a transmission disequilibrium test that allows for locus heterogeneity, and is robust to population stratification^33^. Results of the two stages in the 20p11.22 region (chr20: 21,815,192–21,988,830) were combined using a set association method as implemented in Sumstat^34^ and TDT-HET^33^. The set association method performs multi-locus association by estimating sum statistics for an increasing number of SNPs to evaluate their joint effect on disease. Sumstat and TDT-HET were used because to generate a single *P*-value calculated for the entire region in each study, thereby minimizing the number of tests performed. Previous work has shown that these methods have sufficient power to identify SNPs acting in additive and/or multiplicative manners to increase disease risk^35^. The *P*-value obtained by applying either Sumstat or TDT-HET represents the global significance of the candidate chromosomal region. Combining the two stages in this way yielded increased evidence for association with IS in the 20p11.22 region, with a combined Fisher’s *P*=1.33 × 10^−8^ (Fig. 2 and Table 1) and ORs depicted in Supplementary Fig. 3.

Comparing our results to the National Human Genome Research Institute (NHGRI) GWAS catalogue^22^, we found that the chromosome 20 IS locus was previously associated with early-onset male pattern baldness (AGA). Similar to IS, AGA displays sexual dimorphism, that is, it is biologically unequal in males and females. However, unlike IS, disease progression in AGA (extent of hair loss) is generally more severe in males than in females^36^. We identified chromosome 20p11.22 SNPs that were previously associated with AGA and that were genotyped in our GWAS^37^,^38^,^39^. In this comparison, SNPs that were associated with IS and AGA displayed the opposite direction of effect for the two disorders (Supplementary Table 1). This observation suggested that sequences in the region conferring susceptibility to IS have a protective effect in AGA. To test whether the association we observed was sex-specific, we re-evaluated association with SNPs in the 20p11.22 locus after stratification by sex, that is, separating males and females. This analysis yielded evidence for association with IS in females but not males, with a combined Fisher’s *P*=6.88 × 10^−9^ in the former data set (Table 1 and Supplementary Tables 2 and 3).

To confirm the chromosome 20p11.22 association with IS, we genotyped SNP rs6137473, as it was highly correlated with other top-associated SNPs in the region, in an independent cohort of 216 IS patients ascertained from various paediatric orthopaedic clinics throughout the United States of America and 336 population-matched controls. This variant was significantly associated with IS in females (*P*=2.4 × 10^−4^; OR=1.67, 95% CI=1.27–2.21) but not males (*P*=0.726; OR=0.90, 95% CI=0.49–1.64; Table 2 and Supplementary Table 4). Data provided from a GWAS of female Japanese subjects^24^ also replicated association with the 20p11.22 region (rs6137473 *P*=3.7 × 10^−3^; summary *P*=3 × 10^−3^; Table 2 and Supplementary Table 5). Results from all four studies were combined and confirmed the association of rs6137473 with IS for females (*P*=2.15 × 10^−10^, OR=1.30, 95% CI=1.19–1.41; Table 2).

### Putative enhancers in the chr20p11.22 susceptibility locus

**Figure 3:**
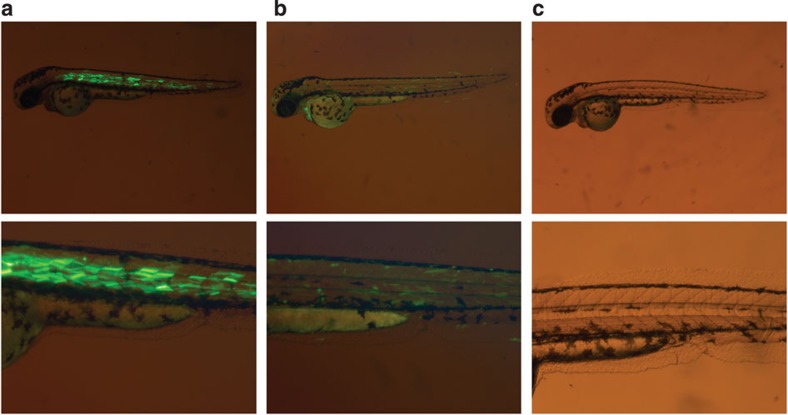
Functional enhancer assays in zebrafish. (**a**) Representative zebrafish (of at least 100 tested for each construct) injected with Xe1-E1b-GFP-Tol2 enhancer construct showing expression predominantly in the somitic muscle. (**b**) Representative zebrafish injected with PE7-E1b-GFP-Tol2 enhancer construct showing expression predominantly in the somitic muscle. (**c**) Representative zebrafish injected with PEC7risk-E1b-GFP-Tol2 enhancer construct leads to loss of expression throughout. Corresponding pictures at higher magnification are shown below.

*PAX1* is a key regulator of sclerotome formation and vertebral development^40^. Naturally occurring missense and deletion mutations in *Pax1* are well-described in the ‘undulated’ and ‘scoli’ mouse strains, so-called because of their distinct tail deformities and varying spinal malformations including scoliosis^40^,^41^,^42^. The orthologous 20p11.22 IS-associated locus overlaps a regulator of murine *Pax1* expression in the developing spine (sclerotome) as identified by murine enhancer trap and reporter assays^43^. In the latter study, deletions of the region including *Pax1* caused a corresponding reduction in gene expression in the sclerotome at embryonic day 11.5 (E11.5). Moreover, an ~1.5-kb sequence ‘Xe1’ encoded in the deleted region recapitulated a *Pax1* expression pattern^43^. We hypothesized that variants in the IS-associated region may affect *PAX1* regulatory elements. Using comparative genomics and ENCODE data for biologically relevant cell lines, human skeletal muscle myoblast and human embryonic stem cells (H1-hESC)^44^, we analysed the associated region (chr20:21,815,192–21,988,830; hg19) for sequences representing putative regulatory elements. We identified ten candidate regions (*PAX1* Enhancer Candidates ‘PEC1’, ‘PEC2’ and so on), including sequences orthologous to the previously described ‘Xe1’ enhancer (Fig. 2 and Supplementary Table 6). To test the ability of each candidate to function as an enhancer, that is, to drive gene expression, we cloned each PEC including Xe1 into the E1b-GFP-Tol2 enhancer assay vector and successfully tested seven of them in zebrafish as previously described^45^. As expected, Xe1 showed enhancer activity in the developing spine, specifically corresponding to somitic muscle (Fig. 3a). One other candidate, PEC7, also displayed functional enhancer activity. Similar to Xe1, PEC7-driven expression of the green fluorescent protein (GFP) reporter was largely restricted to somitic muscle, with weaker expression in spinal cord and heart (Fig. 3b). Thus, Xe1 and PEC7 clearly harboured enhancer activity, possibly in developing spinal muscle, although more detailed studies are needed to precisely define the temporo-spatial effects of these enhancers on gene expression in humans. It is worth noting that PEC7 enhancer expression did not completely overlap the characterized *pax1b* expression in zebrafish at 48 h post-fertilization (h.p.f.)^46^. This could be due to the testing of human sequences in zebrafish. However, previous work has shown that human enhancer sequences can function as active enhancers in zebrafish, even without homologous sequences in zebrafish^47^,^48^,^49^,^50^. In addition, the expression pattern of *pax1a* has yet to be determined in zebrafish, and we cannot exclude the possibility that PEC7 controls the expression of other genes.

### Disruption of enhancer activity by IS-associated SNPs

To search for disease alleles potentially underlying the association with IS, the Xe1 and PEC7 regions were re-sequenced in 48 cases that were enriched for chromosome 20 risk alleles as identified in the original GWAS. Although no variants were detected in Xe1 in these individuals, a haplotype of five variants including top-associated SNP rs169311 was identified in PEC7 (Supplementary Table 7). Using Haploreg^51^, we noted that top SNP rs169311 is predicted to alter binding sites for component of myogenesis protein 1 and vitamin D receptor, and three other PEC7 SNPs are also predicted to alter transcription factor-binding sites (Supplementary Table 7). We compared the ability of the risk haplotype to drive zebrafish reporter gene expression to that of the wild-type sequence. Four independent replicate experiments confirmed that the associated haplotype completely abolished enhancer activity as detected by this assay, suggesting that the IS susceptibility haplotype confers a loss of function for PEC7 enhancer activity (Fig. 3c and Supplementary Data set 1).

### PAX1 expression in spinal myofibers post-somitogenesis

**Figure 4:**
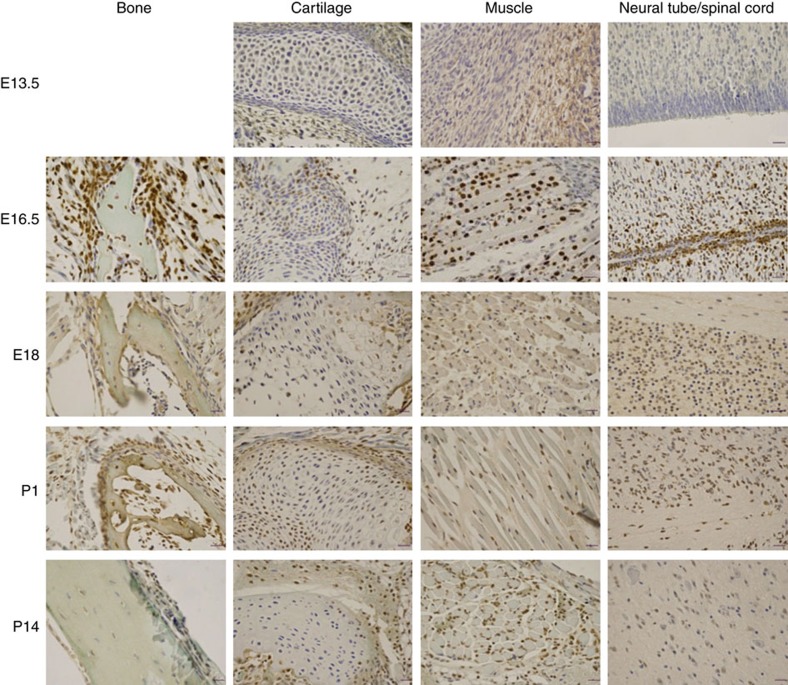
Pax1 immunohistochemistry in developing mouse spinal tissues. Spines and surrounding soft tissue harvested at E13.5, E16.5, E18, P1 and P14 are shown in each row. Representative images for bone, cartilage, muscle and nerve are shown in the columns, with the exception of bone at E13.5, as it was not well-formed at that time point. Muscle was also not well-formed at E13.5 and had a soft tissue appearance. In contrast, strong nuclear staining (brown) was clearly seen in osteoblasts, myoblasts and ependymal cells at E16.5, with weaker chondrocyte staining. More modest staining was evident at E18 and P1 in all tissues. By P14, Pax1 staining persisted in muscle but was essentially absent from bone and cartilage (note positive staining in muscle surrounding cartilage and adjacent to bone). Very weak staining was observed in a subset of spinal cord cells, possibly glia, at P14. Each experimental time point was repeated at least once. Scale bar, 20 μM.

In the developing mouse embryo, Pax1 expression is well-described in somitogenesis, beginning at E8.5 in the stage III somite and continuing to E12.5, becoming restricted to cells surrounding the vertebrae, intervertebral disc anlagen and precursors to the connective tissue around spinal nerve and dorsal root ganglia^40^,^41^. As vertebral structures and segmentation appear normal in IS, we hypothesized a role for PAX1 in spinal development post-somitogenesis. Accordingly, we examined Pax1 protein in mouse spinal tissues at seven developmental time points from E13.5 to postnatal day 84 (P84). As shown in Fig. 4a–c, Pax1 immunohistochemical staining was essentially negative post-somitogenesis at E13.5. However, at E16.5, we observed a striking pattern in developing myofibers, with more modest staining in other cells types. Weaker but persistent staining was evident in myofibers up to stage P84. Pax1 was essentially absent in other spinal cell types (Supplementary Fig. 4). These data demonstrate a potential role for Pax1 in spine development post-somitogenesis, possibly in paraspinous muscles.

## Discussion

We present evidence of a new IS susceptibility locus in an ~100-kb region of chromosome 20p11.22 downstream of *PAX1.* Using a functional fine-mapping approach, we potentially narrow the locus to an ~1.5-kb domain with enhancer activity that is disrupted by disease-associated variants. The *PAX1*-encoding region was originally associated with spinal development through studies of the naturally occurring *undulated* mouse strains. The original *undulated* (*un*) strain, first described in 1947, carries a missense mutation in *Pax1* (ref. 41). *Un/un* mice display a curved spine with malformations of individual vertebrae including the vertebral bodies and intervertebral discs. Three additional strains, *scoliosis* (*sco*) or *undulated intermediate* (un-i), *undulated-extensive* (*un*^*ex*^), *undulated short-tail* (*un*^*s*^), harbour partial or complete deletions of *PAX1*, with the latter including all of the gene and displaying the most severe phenotype^41^,^42^. In early mouse development, Pax1 displays expression restricted to specific structures including the sclerotome that will give rise to the axial spine (vertebrae, ribs, connective tissues and skin). Genomic studies have delineated intervals downstream of *Pax1* harbouring *cis*-regulatory activity consistent with this pattern^43^. In particular, transposon-based deletion mapping and reporter gene assays defined the ~148-kb region 3′ of Pax1 as necessary to drive somitic gene expression (that is, in the dorsal sclerotome) during early mouse development. Furthermore, the mouse Xe1 enhancer encoded in this region was shown to be sufficient to drive a similar expression pattern^43^. Our data using zebrafish transgene assays confirmed the enhancer activity of the human Xe1 orthologue and revealed another element in the region, PEC7, with potential somitic enhancer activity that was disrupted by IS-associated sequence variants. This observation strongly suggests that PEC7 itself functions in IS susceptibility, a hypothesis that may be tested in model systems by targeted mutagenesis.

The spinal anomalies observed in *Pax1* mouse mutants recapitulates a congenital scoliosis phenotype, although mutations in human *PAX1* are not clearly correlated with this condition^52^. By contrast, the structure of the axial spine in IS appears normal. Consequently, we reasoned that either the association we observed reflects functional effects on a gene other than *PAX1*, or an effect on the participation of *PAX1* in a post-somitogenesis developmental role. In support of the latter hypothesis, we found that Pax1 is strongly expressed in developing myofibers of the mouse spine at day E16.5, well after the somites are formed, and that expression persisted at least to early adulthood, albeit at reduced levels. It is intriguing to consider that *PAX1* may participate in multiple roles in spinal development that are dictated by the spatio-temporal control of its expression. Such a scenario predicts that deleterious mutations that occur in *PAX1*-coding sequences, versus those that occur in its regulatory elements, will give rise to distinct but potentially overlapping phenotypes. This phenomenon is well-described for other early developmental genes. For example, coding mutations in the sonic Hedgehog gene produce the severe multi-system disorder holoprosencephaly, whereas mutations in the sonic Hedgehog *cis*-regulator ZRS cause limb-specific pre-axial polydactyly^53^,^54^. Whether congenital scoliosis and IS may be aetiologically connected in humans is unclear but has been suggested from familial aggregation studies^55^,^56^,^57^. An aetiologic relationship between the two diagnoses is also supported by the recent discovery that *ptk7* mutant zebrafish model both IS- and CS-like phenotypes in zygotic and maternal-zygotic genetic backgrounds, respectively^8^. The latter study also illustrates the distinct morphologies that may arise from the differential expression of key developmental genes.

Our investigation of the chromosome 20p11 locus provides the first genetic evidence to explain the puzzling sexual dimorphism that is a hallmark of IS. Besides susceptibility to progression, the pattern, onset and flexibility of deformity also differ between boys and girls^10^. Various hypotheses have been proposed to explain male/female differences in IS, including the existence of X-linked genetic risk factors and effects on circulating hormones. Neither mechanism has been clearly supported, although investigations have been limited^6^,^58^. Our identification of a female-specific IS susceptibility locus suggests an underlying mechanism that is sensitive to the female milieu at the time of adolescence. Although we did not find evidence for oestrogen receptor-binding sites within the PEC7 enhancer locus itself, it is interesting to postulate that this locus increases risk of IS via downstream hormonal interactions. We note in this regard that the next-nearest gene, *FOXA2*, is implicated in sexually dimorphic gene expression via cooperation with androgen and oestrogen receptor^59^. It is possible that PEC7 regulates *FOXA2.* However, we did not detect Foxa2 expression in embryonic or postnatal mouse spine (data not shown) and consider it an unlikely candidate for IS susceptibility. *PAX1* is also expressed in the adult scalp^37^. Whether variants in PEC7 affect this expression and drive association with early-onset male pattern baldness requires further study, but the overlapping genetic association suggests a possible correlation between the two sexually dimorphic conditions.

We provide evidence that *PAX1* is expressed in paraspinal muscles, and to a lesser extent in spinal cord, at time points well after the initial patterning of the axial skeleton has been completed. This suggests a later developmental role for *PAX1*, possibly in the growth or maintenance of these tissues. It is interesting that other IS risk loci identified by GWAS occur in non-coding regions near or within genes involved in muscle and nerve biogenesis. In this regard, we expect that the functional fine-mapping method applied in the present study should prove fruitful for identifying the alleles and functional elements driving association at other IS risk loci. Our results also imply that additional sex-specific genetic loci may await discovery, and draw attention to the need to consider males and females in separate liability classes.

## Methods

### Study subjects

All research subjects included in GWAS I-715, GWAS II and TSRHC III provided written informed consent to participate in the study as approved by the Institutional Review Board of the University of Texas Southwestern Medical Center. For GWAS I and II, patients or former patients were sequentially ascertained in Orthopedic Clinics at TSRHC and enrolled into an idiopathic scoliosis (IS) registry. Parents and other affected family members were also ascertained when possible. For TSRHC III, 216 NHW cases were included from cases ascertained in TSRHC clinics or through collaborating orthopaedic surgeons in the United States of America at Shriners Hospital for Children, Lexington, KY (T. Milbrandt, V. Talwalkar, H.J. Iwinski, R. Muchow); Hasbro Childrens’ Hospital, Providence, RI (C.P. Eberson); University of Massachusetts Memorial Medical Center, Worcester, MA (A. Lapinsky); Childrens Hospital of Wisconsin, Milwaukee, WI (J.C. Tassone, X.C. Liu) and Akron Children’s Hospital, Akron, OH (W. Schrader); OrthoArizona Phoenix, AZ (R. Shindell); Indiana University-Purdue University Indianapolis, Indianapolis, IN, (R. Loder); University of Oklahoma Health Sciences Center, Oklahoma City, OK (J. Davey). All affected subjects in these cohorts met criteria for a positive diagnosis of IS: lateral deviation from the midline greater than 15° as measured by the Cobb angle method from standing spinal radiographs, axial rotation towards the side of the deviation and exclusion of relevant co-existing diagnoses. Blood samples were obtained by venipuncture. In some cases, saliva samples were self-collected using the Oragene DNA kit (DNA Genotek, Inc.). Control individuals were ascertained from within the local Texas population or non-orthopaedic clinics at TSRHC. A diagnosis of scoliosis, or family history of scoliosis, was excluded by questionnaire. To reduce the possibility of biases due to population stratification in the TSRHC III cohort, we previously genotyped 384 ancestry informative markers and performed multi-dimensional scaling analysis of identity-by-state distances to identify outliers^23^.

The Japanese cohort consisted of 1,050 Japanese females with adolescent IS recruited from eight collaborating hospitals (Japan Scoliosis Clinical Research Group: JSCRG) between February 2009 and January 2011. All subjects with adolescent IS underwent clinical and radiologic examinations for IS as previously described^24^. Subjects with definitive family history of Mendelian inheritance were excluded from the study. The control subjects consisted of 1,474 Japanese females, including healthy volunteers from the Midosuji Rotary Club, Osaka, Japan, and individuals who were registered in the BioBank Japan Project but were genotyped in GWAS of other diseases, as previously described^60^. Informed consent was obtained from all Japanese subjects and from parents of minor subjects according to a protocol approved by the ethics committees of the University of Texas Southwestern Medical Center, University of Kentucky, University of Massachusetts Medical School, Children’s Hospital of Wisconsin, Akron Children’s Hospital, Indiana University-Purdue University, Oklahoma University Health Sciences Center and RIKEN.

### GWAS genotyping, quality control, imputation and association testing

The GWAS II data set was derived from 1,201 individuals (457 cases and 744 controls). We chose the sample sizes to have 80% power to detect genetic association with population data (cases and controls) at the 5E-08 significance level (that is, 0.05 genome-wide significance) using the following parameters: genetic model-based design, disease prevalence of 0.03, heterozygote relative risk of 1.5, homozygote relative risk of 2.25, disease allele frequency of 0.50, linear trend test statistic with additive weights (0, 1, 2 for the three possible genotypes at a SNP). Using these specifications, we compute a minimum sample size of 932 cases and 932 controls. The combined Stage I and Stage II cohorts therefore exceeded the sample sizes required for our specified power. DNA samples from cases and controls were distributed non-sequentially into plates and genotyped using the Illumina HumanOmniExpress-12 v1.0 beadchip (Illumina, Inc.) containing 730,498 markers. Twenty-nine technical replicates were included and were 100% concordant. We used PLINK v1.07 (ref. 32) to perform QA on these data. A total of 17 individuals were removed from the analyses. Fifteen had genotyping call rates <95%. Two individuals had ambiguous gender information according to the X-chromosome inbreeding coefficient^61^. SNP markers were evaluated for the presence of heterozygous haploid (HH) genotypes and missingness. All markers on chromosome Y with HH genotypes were removed. Markers on chromosome X with HH genotypes were kept. Markers with more than 5% of missing data were removed. A total of 2,828 markers (0.39%) failed QA. After all QA filters were applied, a total of 1,184 individuals (447 cases and 737 controls) and 727,670 markers were included in the analyses (GWAS II). In addition, we checked for population stratification confounder effects by applying the genomic control (GC) method^62^. We obtained a correction factor (*λ*) of 1.0992, potentially indicating a slight inflation of the GWAS results due to population stratification. To study the region in more detail, we used microarray genotypes to impute 1000 Genomes (http://www.1000genomes.org) chromosome 20 variants with MACH^63^ (http://www.sph.umich.edu/csg/abecasis/MACH/tour/imputation.html). Genotypes were tested for association using CATT as implemented in the PLINK software^32^. Genotyping and quality control for GWAS I-715 were as previously described^23^,^24^. Nine individual samples were removed with genotyping call rates <95%. Of 345,111 SNPs, 1,514 (0.44%) had call rates less than 95% and were removed, leaving 343,597 (99.56%) of SNPs. SNP markers corresponding to X and Y chromosomes were also removed from the analysis. The Japanese GWAS was analysed as described previously^24^,^25^.

Meta-analysis of Stages I and II, TSRHC III and Japan sets assumed a fixed effect model and was carried out using the inverse variance weighting method. Where applicable, meta-analyses of case–control and trio designs were performed as described in Kazeem and Farral^64^.

### Set association method

We applied the set association method, as implemented in the Sumstat^34^ and TDT-HET^33^ software, to the region on chromosome 20 spanning 21,815,192–21,988,830 corresponding to a linkage disequilibrium (LD) block harbouring top-associated SNPs from the second stage GWAS. We used this method to obtain a single global *P*-value for the chromosomal region in each data set (Stage I, Stage II and Japan). Our approach is similar to the analysis of variance approach for testing equality of means across multiple categories. In this analogy, we consider SNPs are categories. We applied TDT-HET to pedigree data from Stage II and Sumstat to case–control data from Stage I and the Japan data set. We obtained three *P*-values this way, each corresponding to a single data set (Stage I, Stage II and Japan). Because each data set was independent, we used Fisher's Combined *P*-value method^65^ to compute a single *P*-value for the chromosomal region.

Association analyses were performed using 100,000 permutations. None of the markers in the selected regions failed either the Hardy–Weinberg proportions test in the unaffected sample or presented significant differences in genotype calling between cases and controls at the 0.1% level. In Supplementary Table 3, the actual permutation *P*-value for the chromosome 20 region in GWAS I-702 (Stage II) was 0. We used the value *P*=3 × 10^−5^ since it is the upper limit of the 95% confidence interval, as determined by the method implemented in the BINOM programme^66^. An interesting result from our association analyses is that we observed a *P*-value of 0.19 in Stage II pedigree data (affected subjects restricted to females) for the single marker rs6137473. When we applied the TDT-HET method to the markers in the candidate region for the same pedigrees, our multi-locus *P*-value became highly significant. In addition, *P*-values obtained with female population data are also highly significant for the same marker. We conjecture that this result is due to differences in sample size, especially given that, for family-based association, trios must contain at least one heterozygous parent to be informative. When we checked the number of trios with female-only affected subjects for marker rs6137473, we computed a total of 201 informative trios out of the total 600. We performed comparative power calculations for case–control females, and for trios. We used parameter settings from both Table 1 and Table 2. Using these values, we computed a power value of 70% for the case–control design using the linear trend test (weights equal to number of disease alleles in respective genotype) at a 10^−5^ significance level. On the other hand, we computed a power of 12% for the TDT design at the same significance level. This decrease in power is substantial, and provides one possible explanation as to why we did not observe a clear replication with the TDT statistic at the single-locus level.

### TSRHC III genotyping

We genotyped SNP rs6137473 in the 216 unrelated cases and 336 unrelated controls using Taqman genotyping. Results were in agreement with Hardy–Weinberg equilibrium (*P*>0.05). We performed allelic tests of association and CATT as implemented in the PLINK software^32^.

### Candidate enhancer identification

Using the UCSC Genome Browser (https://genome.ucsc.edu/cgi-bin/hgGateway), PhastCons (http://compgen.bscb.cornell.edu/phast/help-pages/phastCons.txt) conserved elements and PhyloP (http://compgen.bscb.cornell.edu/phast/help-pages/phyloP.txt) scores, we analysed chr20:21,815,192–21,988,830 (hg19) for evolutionary conserved sequences. In addition, we used enhancer mark data (H3K27ac and H3K4me1) from the ENCODE project for human skeletal muscle myoblast cells and human embryonic stem cells (H1-hESC), as well as DNase hypersensitivity data to identify putative regulatory elements within the chromosome 20 region. These two methods of analysis led to the selection of ten regions that were conserved between human and other placental mammals or were predicted regulatory elements based on ENCODE data.

### Re-sequencing study

To identify individuals for re-sequencing we used the top Stage I CATT-associated SNP markers on chromosome 20 (rs6137473, rs6106434, r169311). Two of these SNPs (rs6106434 and rs169311) flank the Xe1 enhancer region. We determined all possible genotype patterns of the three markers. To prioritize cases and controls for re-sequencing, we selected the multi-locus genotype pattern (MLGP) that (i) showed the largest difference in frequency between cases and controls, (ii) had at least five samples in cases and controls and (iii) for which the frequency in cases was greater than in controls. The MLGP that met these criteria was GG/AA/AA, corresponding to the homozygous minor alleles at rs6137473, rs6106434 and rs169311, respectively. Forty-eight NHW IS cases with this MLGP were selected for re-sequencing. PCR primers were designed to flank PEC7 and Xe1 regions. PCR fragments were sequenced using the Sanger method.

### Zebrafish assays

Male and female strain AB zebrafish were used in all experiments. Enhancer candidate sequences were cloned by PCR from human genomic DNA (Roche) into the E1b-GFP-Tol2 enhancer assay vector containing an E1b minimal promoter followed by GFP^67^ and verified by sequencing. For the PEC7 risk haplotype, PCR was carried out on an individual encompassing this haplotype and sequence verified. Each construct was injected at two different days and Xe1, PEC7-reference and PEC7 risk haplotype at four different days following standard procedures^68^,^69^ into at least 100 zebrafish embryos along with Tol2 mRNA^70^ to facilitate genomic integration. GFP expression was observed and annotated up to 48 h.p.f using a Leica M165 FC microscope. An enhancer was considered positive if at least 10% of all fish surviving to 48 h.p.f. showed a consistent expression pattern after subtracting out percentages of tissue expression in fish injected with the empty enhancer vector. For each construct, at least 100 fishes were analysed for GFP expression up to 48 h.p.f. All animal work was approved by the University of California San Francisco Institutional Animal Care and Use Committee.

### Pax1 immunohistochemistry

All animal work was approved by the University of Texas Southwestern Medical Center Institutional Animal Care and Use Committee. Male and female *BL6* mice were euthanized at different developmental stages (E13.5, E16, E18, P1, P14, P28, P84) by asphyxiation with CO_2_. Spines were excised and fixed with 10% buffered formalin for 3 days and then processed in a Leica ASP300S tissue processor and embedded in paraffin. Four-micrometre sections were cut in series in the frontal plane and loaded onto 3-aminopropyltriethoxysilane-coated slides. Antigen retrieval was performed with 0.25% Trypsin (Sigma-Aldrich) and endogenous peroxidase was blocked with 3% H_2_O_2_ (Thermo Fisher Scientific Inc.). Slides were incubated with rabbit polyclonal antibody (1:15,000, Sigma-Aldrich catalogue number SAB2101727) directed against a PAX1-specific peptide and detected by goat anti-rabbit IgG-HRP (1:500, EMD Millipore Corporation) and 3,3′-diaminobenzidine (Dako) as chromogenic substrate. After Pax1 staining, sections were counterstained with haematoxylin and Fast Green (Sigma-Aldrich) and mounted with Cytoseal XYL (Thermo Fisher Scientific Inc.). Haemotoxylin and eosin staining were also performed separately for pathological characterization. Slides were viewed with an Olympus BX40 microscope.

## Additional information

**How to cite this article:** Sharma, S. *et al*. A *PAX1* enhancer locus is associated with susceptibility to idiopathic scoliosis in females. *Nat. Commun.* 6:6452 doi: 10.1038/ncomms7452 (2015).

## Supplementary Material

Supplementary Information Supplementary Figures 1-4 and Supplementary Tables 1-7

Supplementary Dataset 1 Zebrafish enhancer assay results. Relative GFP expression, calculated by subtracting raw GFP from background (empty vector), are highlighted in green.

## Figures and Tables

**Table 1:** GWAS summary statistics.

	**Stage I**			**Stage II**			**Combined**	
	***N*** **(cases/controls)**	***P*** **-value**	**OR**	**Trios (subjects)**	***P*** **-value**	**OR**	***P*** **-value**	**OR**
Total	447/737	2 × 10^−5^	1.44	715 (1,876)	3 × 10^−5^*	1.23	1.33 × 10^−8^	1.31
Females	371/533	1 × 10^−5^	1.54	600 (1583)	3 × 10^−5^*	1.21	6.88 × 10^−9^	1.35
Males	76/204	0.631	1.08	115 (293)	0.543	1.25	0.71	1.15

GWAS, genome-wide association study; OR, average odds ratio.

Summary statistics obtained using Sumstat^34^ and TDT-HET^33^ were applied to SNPs in the chromosome 20 region spanning genomic positions 21,815,192 to 21,988,830 in both data sets. Combined *P*-values were calculated Fisher’s Combined *P*-value method^65^.

^*^Actual permutation *P*-value was 0. The value in the table is the upper bound of the 95% confidence interval determined by the BINOM programme.

**Table 2:** rs6137473 association results in all data sets.

**Study**	**Risk allele**	**Females**		**Males**		**Combined**
		***P***	**OR (95% CI)**	***P***	**OR (95% CI)**	***P***
Stage I	G	2.36 × 10^−7^	1.64 (1.36–1.98)	0.67	1.09 (0.75–1.58)	5.58 × 10^−7^
Stage II	G	0.19	1.16 (0.93–1.45)	0.45	1.21 (0.74–1.97)	0.13
TSRHC III	G	2.40 × 10^−4^	1.67 (1.27–2.21)	0.73	0.90 (0.49–1.64)	1.50 × 10^−3^
Japan	G	3.70 × 10^−3^	1.18 (1.06–1.33)	NA	NA	NA
Combined		2.15 × 10^−10^	1.30 (1.19–1.41)	0.81	1.08 (0.82–1.41)	3.12 × 10^−8^

CI, confidence interval; NA, data not available; OR, odds ratio; TSRHC, Texas Scottish Rite Hospital for Children.

Results of allelic association tests performed using Cochran Armitage trend test are shown for the case–control studies (Stage I, TSRHC III and Japan). TDT-HET *P*-values are shown for the family-based study (Stage II).
