# Supplementary material for: A PAX1 enhancer locus is associated with susceptibility to idiopathic scoliosis in females
Source: Nat Commun. 2015 Mar 18;6:6452. doi: 10.1038/ncomms7452 (PMC4365504; doi:10.1038/ncomms7452)
Supplement: Supplementary Information — Supplementary Figures 1-4 and Supplementary Tables 1-7 [file ncomms7452-s1.pdf]

Supplementary Figures and Tables

Genotyped association

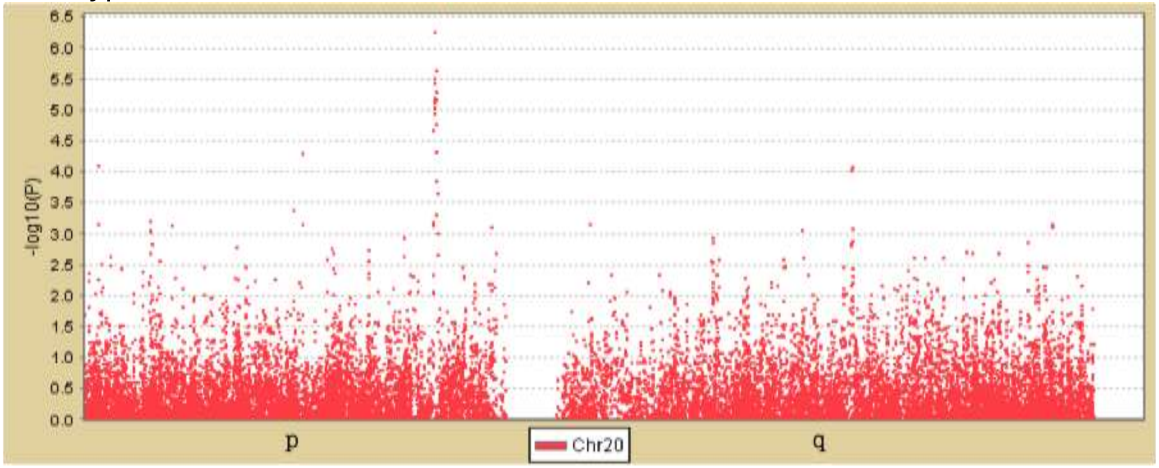

Imputed association

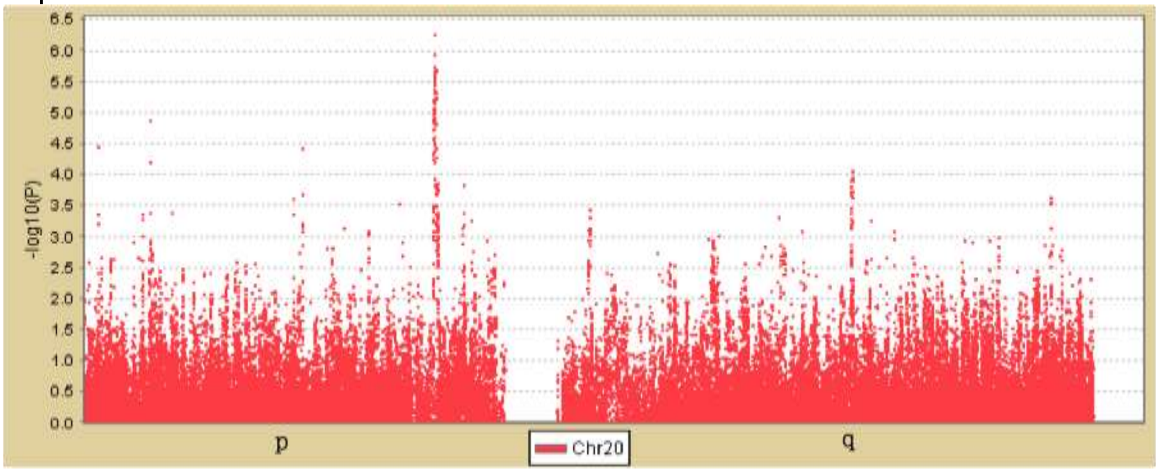

Supplementary Figure 1. Association plots of chromosome 20 SNPs in the stage I GWAS. Association CATT p-values are plotted versus chromosomal position for genotyped SNPs (top), and including imputed SNPs (bottom).

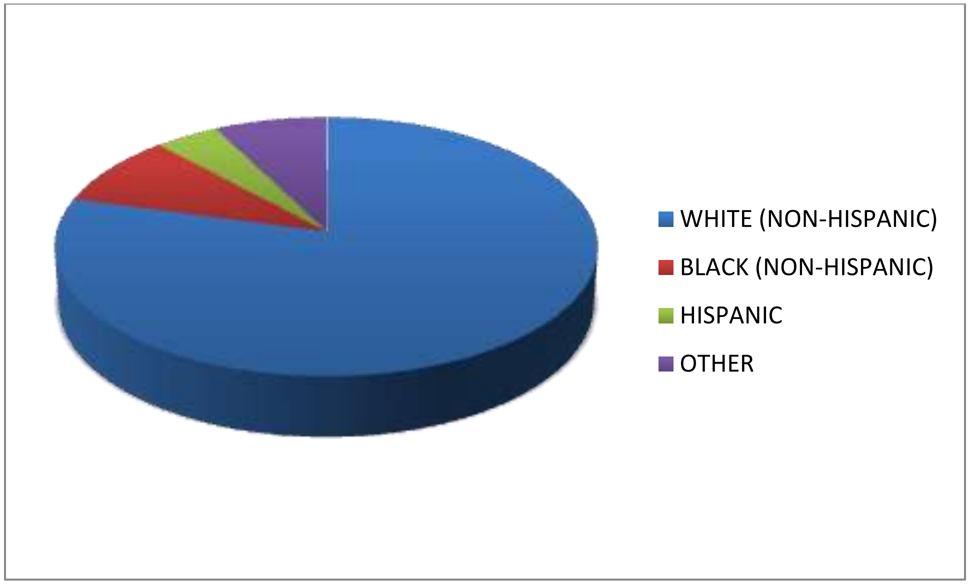

Supplementary Figure 2. GWASI-715 composition. Relative proportions (of 715 probands total) for four ancestral groups are shown by color.

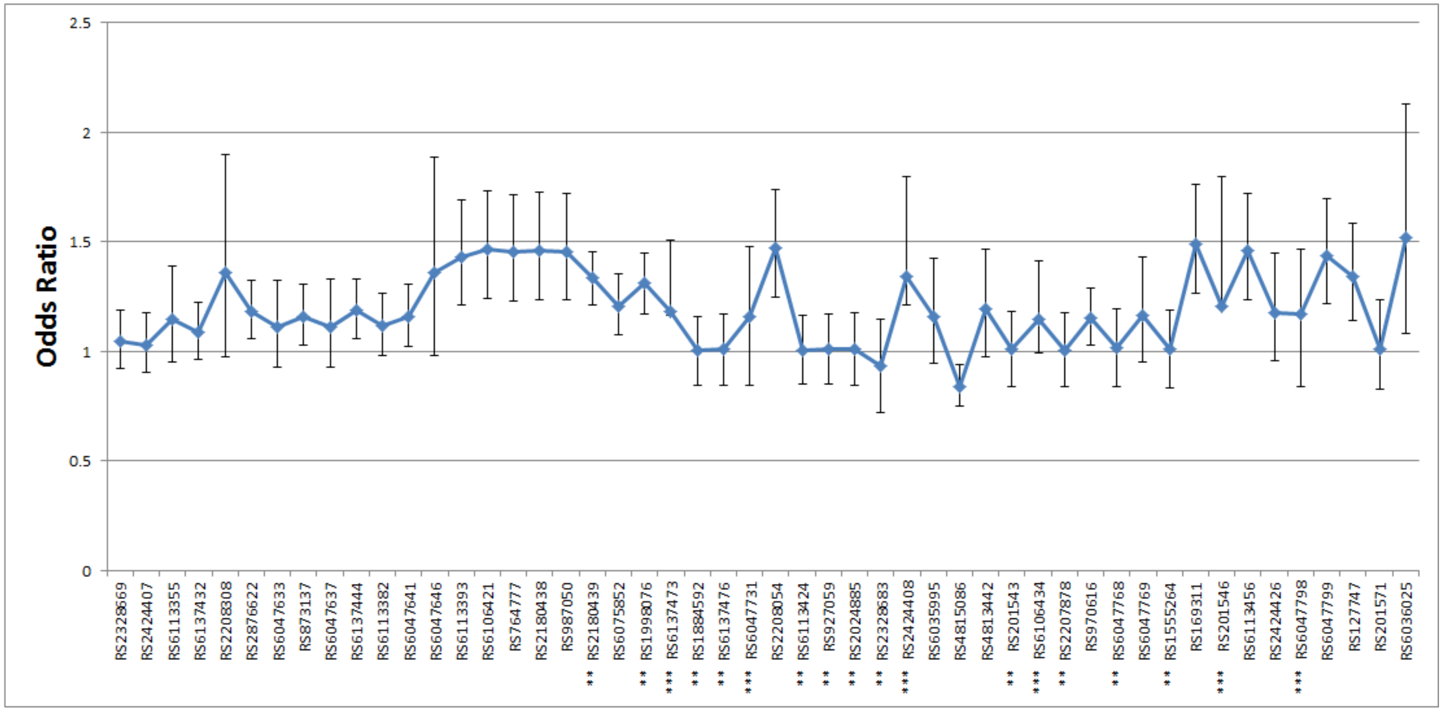

Supplementary Figure 3. Minimum, maximum and median odds ratio (OR) for each SNP in the selected chromosomal region across all GWAS datasets (GWASII, GWASI-715, Japan GWAS). The upper and lower values of the error bars represent the upper and lower limits of the 95% OR confidence interval estimated when the marker was present in only one of the datasets. If the marker was present in more than one dataset (denoted by asterisks), the error bars represent the maximum and minimum ORs.

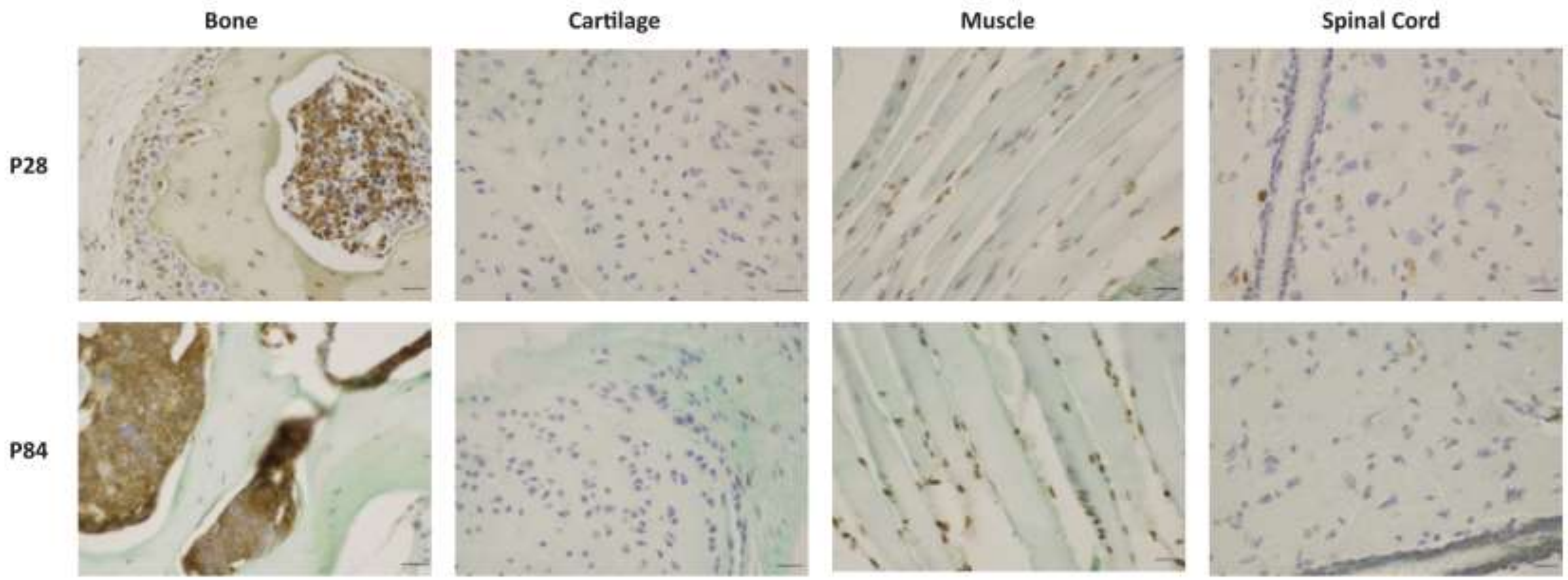

Supplementary Figure 4. Pax1 immunohistochemistry in developing mouse spinal tissues. Spines and surrounding soft tissues were harvested at postnatal days 28 and 84 (P28, P84) are shown in each row. Representative images for bone, cartilage, muscle, and nerve are shown in the columns. Strong positive nuclear staining persisted in muscle cells, but was much weaker and less consistent in bone, cartilage, and spinal cord. Dark brown patches in bone are due to infiltrating blood cells.

			AGA		IS (GWASII)		
**SNP**	**Position (hg19)**	**Alleles**	**Risk Allele**	**Reference**	**Risk Allele**	**P-value***	**OR (95% CI)**
rs2180439**	21,872,462	C/T	T	17	C	5.69×10^{-6}	1.47 (1.24 - 1.74)
rs6137444	21,805,001	C/T	T	17	C	3.18×10^{-4}	1.36 (1.15 - 1.61)
rs1998076**	21,899,407	A/G	G	17	A	8.74×10^{-6}	1.46 (1.23 - 1.72)
rs201571	22,032,876	C/T	T	17	C	1.43×10^{-4}	1.38 (1.17 - 1.63)
rs6113491	22,076,777	A/C	A	17	C	1.82×10^{-4}	1.30 (1.10 - 1.54)
rs1160312	22,069,865	G/A	A	18	G	9.20×10^{-4}	1.33 (1.13 - 1.56)
rs913063	22,061,780	C/A	A	18	C	7.52×10^{-4}	1.33 (1.13 - 1.57)
rs6047844	22,056,937	C/T	T	19	C	1.43×10^{-4}	1.38 (1.17 - 1.63)

Supplementary Table 1. SNPs in the 20p11 IS susceptibility region associated with early onset androgenic alopecia (AGA). OR = odd ratios; (95% CI) denotes the lower and upper bounds of the 95% confidence interval; *denotes trend test results; **denotes SNPs genotyped in Stage I (GWASII); other SNP genotypes were imputed.

SNP	Position (hg19)	Alleles	Risk allele	FEMALES P	FEMALES OR (95% CI)	MALES P	MALES OR (95% CI)
rs6113393	21815192	A/G	G	1.92×10^{-5}	1.51 (1.25 - 1.82)	0.46	1.15 (0.79-1.67)
rs6106421	21821217	A/G	G	6.41×10^{-6}	1.54 (1.28 - 1.86)	0.35	1.19 (0.82-1.74)
rs764777	21823771	A/G	A	7.89×10^{-6}	1.53 (1.27 - 1.85)	0.35	1.19 (0.82-1.74)
rs2180438	21849282	A/C	A	5.40×10^{-6}	1.55 (1.28 - 1.87)	0.43	1.16 (0.80-1.69)
rs987050	21852049	A/C	A	7.89×10^{-6}	1.53 (1.27 - 1.85)	0.43	1.16 (0.80-1.69)
rs2180439	21853100	A/G	G	8.36×10^{-6}	1.53 (1.27 - 1.85)	0.43	1.16 (0.80-1.69)
rs1998076	21880045	A/G	A	9.60×10^{-6}	1.53 (1.26 - 1.84)	0.52	1.13 (0.78-1.65)
rs6137473	21884693	A/G	G	2.37×10^{-7}	1.64 (1.36 - 1.99)	0.66	1.09 (0.75-1.58)
rs6047731	21899141	A/G	G	8.56×10^{-7}	1.61 (1.33 - 1.94)	0.65	1.09 (0.75-1.58)
rs2208054	21899708	A/G	A	1.17×10^{-6}	1.60 (1.32 - 1.93)	0.65	1.09 (0.75-1.58)
rs2328683	21918447	A/G	A	5.60×10^{-5}	1.47 (1.22 - 1.78)	0.69	1.08 (0.74-1.57)
rs2424408	21924173	A/G	A	2.31×10^{-2}	1.48 (1.05 - 2.09)	0.31	1.41 (0.73-2.78)
rs6106434	21949593	A/G	A	8.29×10^{-5}	1.46 (1.21 - 1.77)	0.36	1.19 (0.81-1.76)
rs169311	21962333	A/G	A	8.10×10^{-7}	1.61 (1.33 - 1.94)	0.54	1.12 (0.77-1.63)
rs201546	21967950	A/G	A	2.84×10^{-2}	1.46 (1.04 - 2.06)	0.31	0.71 (0.36-1.38)
rs6113456	21977094	A/C	C	4.19×10^{-6}	1.56 (1.30 - 1.89)	0.53	1.12 (0.77-1.63)
rs6047798	21985498	A/G	A	5.72×10^{-6}	1.56 (1.29 - 1.88)	0.40	1.17 (0.81-1.71)
rs6047799	21986312	A/C	A	8.20×10^{-6}	1.54 (1.28 - 1.86)	0.66	1.09 (0.75-1.58)
rs127747	21988830	A/G	A	8.76×10^{-5}	1.46 (1.21 - 1.77)	0.97	0.99 (0.68-1.44)
				**SUMSTAT P = 0.00001**	**AVG = 1.54**	**SUMSTAT P = 0.63**	**AVG OR = 1.08**

Supplementary Table 2. Tests of association for SNPs in the chr20p11.22 candidate region for Stage I females and males. Tests for association with IS using the Cochran-Armitage Trend Test (CATT) are shown. OR = odds ratio; (95% CI) = lower and upper bounds of the 95% confidence interval.

SNP	Position (hg19)	Alleles	Risk allele	FEMALES P	FEMALES OR (95% CI)	MALES P	MALES OR (95% CI)
rs1998076	21880045	A/G	A	0.18	1.17 (0.93-1.47)	0.45	1.21 (0.74 - 1.97)
rs6137473	21884693	A/G	G	0.19	1.16 (0.93-1.45)	0.45	1.21 (0.74 - 1.97)
rs1884592	21890690	A/G	G	0.20	1.16 (0.93-1.44)	0.53	1.17 (0.71 - 1.92)
rs6137476	21897656	A/G	A	0.20	1.16 (0.93-1.40)	0.39	1.24 (0.76 - 2.02)
rs6047731	21899141	A/G	G	0.20	1.16 (0.93-1.44)	0.53	1.17 (0.71 - 1.92)
rs6113424	21906780	A/G	G	0.18	1.16 (0.93-1.45)	0.53	1.17 (0.71 -1.92)
rs927059	21914194	A/G	A	0.18	1.16 (0.93-1.45)	0.45	1.21 (0.74 - 2.00)
rs2024885	21917393	A/G	A	0.18	1.16 (0.93-1.45)	0.38	1.24 (0.76 - 2.02)
rs2328683	21918447	A/G	A	0.34	1.11 (0.89-1.39)	0.26	1.32 (0.81 - 2.16)
rs2424408	21924173	A/G	A	0.006	1.79 (1.18-2.73)	0.22	1.83 (0.68 - 4.96)
rs6035995	21931683	A/G	G	0.19	1.16 (0.93-1.45)	0.53	1.17 (0.71 - 1.92)
rs4813442	21939656	A/G	A	0.13	1.19 (0.95-1.48)	0.39	1.24 (0.76 - 2.02)
rs201543	21946928	A/G	A	0.14	1.18 (0.95-1.48)	0.45	1.21 (0.74 - 1.98)
rs6106434	21949593	A/G	A	1	1.00 (0.80-1.25)	0.89	0.97 (0.58 - 1.62)
rs2207878	21951847	A/G	G	0.16	1.17 (0.94-1.46)	0.46	1.20 (0.74 - 1.95)
rs6047768	21953649	A/G	G	0.11	1.20 (0.96-1.49)	0.46	1.20 (0.74 - 1.95)
rs6047769	21958049	A/G	A	0.19	1.16 (0.93-1.45)	0.45	1.21 (0.74 - 1.97)
rs1555264	21961410	A/G	G	0.14	1.18 (0.95-1.47)	0.38	1.24 (0.76 - 2.02)
rs201546	21967950	A/G	A	0.006	1.79 (1.18-2.73)	0.22	1.83 (0.68 - 4.96)
rs2424426	21980725	A/G	G	0.15	1.18 (0.94-1.48)	0.59	1.15 (0.68 - 1.95)
rs6047798	21985498	A/G	A	0.13	1.19 (0.95-1.49)	0.71	1.10 (0.67 - 1.80)
rs201571	22013514	A/G	G	0.87	1.02 (0.89-1.27)	0.89	0.97 (0.57 - 1.62)
rs6036025	22039868	A/G	A	0.025	1.53 (1.05-2.23)	0.34	1.46 (0.68 - 3.13)
**Global**				**P = 0**	**AVG OR = 1.21**	**P = 0.54**	**AVG OR = 1.25**

Supplementary Table 3. Tests of transmission disequilibrium for SNPs in the chr20p11.22 candidate region for Stage II females and males. The global TDT-HET p-value is the same order of magnitude as the SumStat global p-value for the stage I data, while the stage II single marker p-values are generally non-significant at the 0.05 level. It is interesting that the global TDT-HET p-value is more significant that any single-locus p-value. We conjecture that this result may be due to the fact that set association statistics like TDT-HET can be more powerful than single locus procedures when the loci act in an additive fashion to confer disease risk[1,2].

	Risk allele	Risk allele frequency in cases	Risk allele frequency in controls	P	OR (95% CI)
**FEMALES**	G	0.52	0.39	0.0002	1.67 (1.27 - 2.21)
**MALES**	G	0.41	0.44	0.73	0.90 (0.49 - 1.64)

Supplementary Table 4. Test of association for SNP rs6137473 in females and males in TSRHC III.
rs6137473 is located at position chr20: 21884693 (hg19).

SNP	Position (hg19)	Alleles	Risk allele	Risk allele frequency in cases	P	OR (95% CI)
rs2180439*	21853100	C/T	C	0.42	9.15E-04	1.22 (1.08 - 1.36)
rs6137473	21884693	A/G	G	0.48	3.48E-03	1.19 (1.06 - 1.33)
rs6047731*	21899141	C/T	C	0.54	3.68E-03	1.18 (1.06 - 1.32)
rs2424408*	21924173	C/T	T	0.04	7.62E-02	1.35 (0.97 - 1.86)
rs6106434	21949593	A/G	A	0.50	1.60E-02	1.15 (1.03 - 1.29)
rs201546*	21967950	C/T	T	0.12	3.77E-02	1.21 (1.01 - 1.45)
rs6047798	21985498	A/G	A	0.54	2.87E-03	1.19 (1.06 - 1.33)
					**SUMSTAT P = .0038**	**AVG OR = 1.21**

Supplementary Table 5. CATT association results for SNPs in the chr20p11.22 candidate region for females in the Japan study. *Risk alleles differ from GWASII and GWASI-715 due to strand differences of the different Illumina beadchips. Hence rs2180439 and rs6047731 C in the Japan study corresponds to G in GWASII and GWASI-715, rs2424408, rs201546 T in the Japan study corresponds to A in GWASII and GWASI-715.

PEC number	Chr 20 region (hg19)	SNP	P-value	Forward Primer	Reverse Primer
1	21,817,778-21,819,919			TGGTGATGCTCTGGATTCAA	GTACTCACCTCACGCAGCAA
2	21,916,798-21,919,272	rs2328683	5.94×10^{-5}	TTTTGCCTACAGCTGCTTTG	GATCCACGATGAGTGGGAGT
3	21,843,803-21,845,771			CCTACACCTCAGCCTCAAGC	CTGGTCTTCAGTGCCCTTTC
4	21,881,024-21,882,990	rs8122537**	0.719	TTTGTAGGGACTGGGTCCTG	GACTTGAAATGTTCCCAGCA
5	21,916,951-21,919,161	rs2328683	5.94×10^{-5}	TTTTGCCTACAGCTGCTTTG	AGAAGGCAACGGCATTCTAA
6	21,951,501-21,952,876			CTGGAAATGCCACCTCTGAC	TTATGACCACCCTCCCAAAA
Xe1	21,958,600-21,960,008	rs6106437*	NA	GTGGGGGAAAATTGCTTTTC	GAACCAGTTGGGGATGAAGAG
7	21,961,045-21,964,227	rs169311	7.82×10^{-7}	CCCCCTACTTTTCCAGATCC	GGAGACAGCAGGGCTAGATG
8	21,984,667-21,986,714	rs6047798	4.05×10^{-6}	GCTTGTGCAAATCATGCAAC	GGGCTGGCACAGAATTTTTA
9	21,900,610-21,902,880			ATCTCATTGGCCAAACCAAG	ATGATGCCTGTCTCCCTCAT
10	21,897,591-21,899,219	rs150442048<br>rs6047731*	NA<br>NA	TGTTCACTAATCGGGCTCCTATG	CATTCGTCTCTCACCCATGAG

Supplementary Table 6. PAX1 candidate enhancer (PEC) locations. SNPs contained in any PEC are shown along with association P-value in the discovery GWAS. Primers for sequencing and cloning PEC fragments are given. * not genotyped ** low frequency SNP (MAF= .0006)

SNP marker	Position (hg19)	Alleles	IS Genotype	IS Genotype Frequency	1000G CEU Genotype Frequency	Disrupted TF binding site
rs11699227	21,961,920	C/T	CC	1.00	0.259	TATA
rs6036003	21,961,964	A/G	AA	1.00	0.259	4 altered motifs:HNF4, RAR, RXRA, STAT
rs169311	21,962,333	A/C	AA	1.00	0.259	4 altered motifs: BATF; COMP1, Irf, VDR
rs201545	21,962,422	A/C	CC	1.00	1.000	N/A
rs5840940	21,962,533	-/T	-/T	1.00	0.329	N/A
rs2424421	21,963,058	C/T	CC	.98*	0.259	10 altered motifs: Cart1, Foxa, Foxp1, GATA, HDAC2, Hmx_2, Irf, Pax-5, RXRA, P300

Supplementary Table 7. PEC7 re-sequencing in 48 "high risk" idiopathic scoliosis cases. Annotated SNPs, hg19 position, and genotypes are given. Frequencies for the same genotype in CEU (CEPH Utah residents of northern and western European ancestries) are given for comparison. *one sample (of 48) carried C/T genotype. Abbreviations: 1000G - 1000 genomes; TATA - TATA box; HNF4 - hepatocyte nuclear factor 4; RAR - retinoic acid receptor; RXRA - RXR heterodimer binding site; STAT - signal transduction and activator of transcription; BATF - basic leucine zipper transcription factor, ATF-type; COMP1 - cooperates with myogenic proteins 1; VDR - vitamin D receptor; Cart1 - Cartilage homeoprotein 1; Foxa - Forkhead box a; Foxp1 - forkhead box protein 1; GATA - GATA binding factor; HDAC2 - histone deacetylase 2; Hmx_2 - H6 family homeobox 2 (inner ear and vestibular function); Irf - interferon regulatory factor; Pax-5 - PAX5 binding site; P300 - transcriptional co-activating protein

References

1 Hoh, J., Wille, A. & Ott, J. Trimming, weighting, and grouping SNPs in human case-control association studies. Genome research 11, 2115-2119, doi:10.1101/gr.204001 (2001).

2 Hoh, J. & Ott, J. Mathematical multi-locus approaches to localizing complex human trait genes. Nat Rev Genet 4, 701-709, doi:10.1038/nrg1155 (2003).
